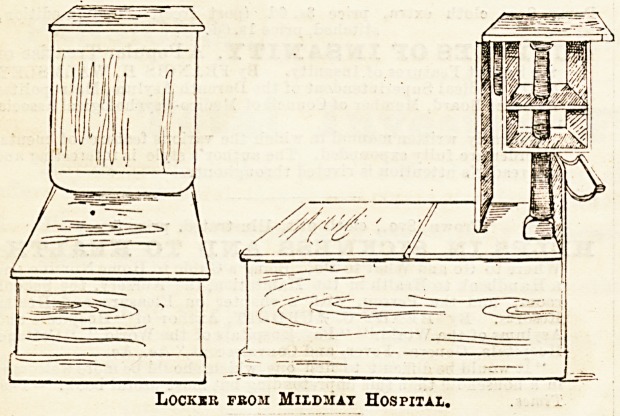# The Hospital Nursing Supplement

**Published:** 1895-11-09

**Authors:** 


### "The Hospital" Nursing Mirror.

Being the Extra Nursing Supplement of "The Hospital" Newspaper.

[Contributions for this Supplement should be addressed to the Editor, The Hospital, 428, Strand, London, W.C., and should have the word "Nursing" plainly written in left-hand top corner of the envelope.]

### News from the Nursing World.

### PRINCESS BEATRICE AT EDINBURGH.

The opening of the Royal Edinburgh Hospital for Sick Children by Princess Beatrice last week was made the occasion of a very large and pleasant assemblage. In addition to the ticket-holders admitted to the building, thousands of spectators waited outside the hospital to see the popular Princess. A guard of honour was provided by the City Garrison, and the scene, over which the Queen's colours floated, was a particularly gay one. Her Majesty became patron of the hospital in 1863, and thirty years later the original premises were condemned as utterly inadequate. A new site was secured in 1893, and the present beautiful building has since been erected. In the course of her inspection of the wards, in which 120 patients can be accommodated, Her Royal Highness, at the request of Dr. Joseph Bell, presented each nurse with a badge in memory of her visit. This consisted of the City arms in silver with the name of the institution, fastened by a ribbon of the Princess of Wales' Danish colours. The badges were provided by the four physicians, and one in gold was handed to the Lady Superintendent, Miss Piggott.

### VISITORS AT WESTMINSTER HOSPITAL.

A great many old friends met on the occasion of the re-opening by the Duke of Westminster of Westminster Hospital last week, large throngs of guests inspecting each department, and the recent additions and improvements are dealt with in another column. The nurses' pleasant sitting-room was appreciated by the numerous visitors who were entertained there, and the matron, Miss Pyne, the Home-sister, and the other members of the nursing staff were indefatigable in looking after the strangers, who could not fail to carry away favourable impressions of the kindly courtesy pervading the fine old hospital.

### THE TRAINED NURSES' CLUB.

The exhibition of nursing appliances which has been held by the editors of *Nursing Notes* at 12, Buckingham Street, has been a most complete success. The number of visitors each day has ranged from seventy to ninety, and nurses from every hospital in London and from many other institutions have eagerly availed themselves of this opportunity for seeing appliances of modern invention. But the exhibition has also served a secondary useful purpose by attracting many new friends to the club in whose rooms it was held. This "Trained Nurses' Club" has gone on its quiet way for many years, valued by its members, but comparatively unknown to other nurses. For a most moderate subscription, the use of the library and club rooms can be enjoyed, and members can meet their friends and have tea at this convenient and central spot. All information can be obtained from Mrs. Nichols, the hon. secretary.

### NURSES AT WORCESTER INFIRMARY.

The urgent need for a nurses' home at Worcester Infirmary is self-evident from a statement of the chairman reported in the local press that at present the nurses are worse off than the laundry and ward maids. A convenient site for a suitable building now seems to be available, and the chairman suggests that the home should contain accommodation for forty nurses, twenty-five required for the infirmary, the rest being private nurses. Cordially agreeing with the suggestions as to the value of a permanent staff of private nurses to the general public, we disagree entirely with the proposition that these workers should prove a source of income to the institution. They must receive proper fees for their services, and be boarded and lodged comfortably between their cases, and any profit on their earnings remaining over should be devoted to pensions for old age and sickness.

### NURSES AS STALL-HOLDERS.

A very wholesome objection appears to exist in Bristol against the misuse of nurses' uniforms, a medical contemporary relating that condemnation has been heard on all sides respecting the hiring of a stall by the Children's Hospital Committee at the Handicrafts Exhibition. On this stall appliances, bandaged dolls, and pamphlets were exhibited by the nurses, who, however, at length protested so seriously against their novel duties that they were permitted to withdraw. Presumably the committee failed to see that they put their nurses on a level with untrained women who sell proprietary drugs, or pose in other public positions in uniform. It is to be regretted that the popularity of a nurse's dress also leads to its being copied at fancy bazaars by young ladies whose efficiency as saleswomen does not condone their coquettish travesties of professional garments. Nurses themselves are always willing to give their services at work sales held for and in their own hospitals, but we certainly sympathise with them in their objections to being used at Bristol or elsewhere for advertising purposes.

### YOUNG WOMEN'S CHRISTIAN ASSOCIATION.

The London Young Women's Christian Association, which has its headquarters at 16a, Old Cavendish Street, has a varied programme of evening lectures and educational classes. Full particulars can be obtained on application to the secretary, Mr. Kidner. Lectures, with lime-light illustrations, form the novel feature of the present season, whilst hygiene, musical drill, sewing, dressmaking, cookery, music, and many other things are taught at the various centres. Amongst the affiliated institutions, Miss Ely's Home for Private Nurses, 25, Norfolk Crescent, is mentioned, and the "Nurses' Union," of which Adeline, Duchess of Bedford, is president, and Miss D. Ashwood, 39, Bryanston Square, secretary.

### NOTTINGHAM CHILDREN'S HOSPITAL.

The Children's Hospital at Nottingham contains accommodation for thirty children, who are admitted up to the age of thirteen years. Besides the sick wards there is a play-room for convalescents. A comfortable carriage, in which the little invalids go for country drives, is a memento of a generous donor. Another liberal friend of the hospital has for years past provided a convalescent home during three or four summer months. The hospital is exceptionally fortunate in possessing such auxiliaries, and its present need of a larger maintenance fund will surely be generously responded to. Probationers are taken for two years' training, and receive a certificate.

### THE COLNEY HATCH FLYLEAF.

The monthly Flyleaf which records the asylum news of Colney Hatch gives a list of the services held in the chapel and a brief chronicle of recent events. Amongst the latter is the harvest festival held in October and also a welcome gift from Messrs. Smith of some two hundred volumes for the library. Items of news about former workers form the feature doubtless chiefly appreciated by the attendants and nurses of the Colney Hatch Asylum.

### WEST RAINTON DISTRICT NURSE.

Nurse Cameron, whose work amongst the sick during the time she has been attached to the West Rainton Nursing Association has won for her the esteem and affection of all in the district, recently resigned her appointment to take up other duties. At a meeting held previous to her departure, Nurse Cameron was cordially thanked for her invaluable services, and was presented with a gold watch and brooch, which had been subscribed for by many friends. Nurse Rudd has accepted the post left vacant by Nurse Cameron's retirement.

### BENWELL NURSE SOCIETY.

The Benwell Nurse Society for the Sick has issued a satisfactory report of a year's work, Nurse Cleobury having paid 1,354 visits to 119 patients. The ladies on the committee have also provided the latter with milk, beef tea, jelly, &c., and other friends have sent clothes. The balance sheet is equally satisfactory, for it shows a small sum in hand, and it is pleasant to note a donation of £5 6s. 6d. from the pitmen at Benwell Colliery. It is evident that the work done by the Nurse Society is heartily appreciated by doctors and patients, and other associations would do well to imitate Benwell in granting a proper allowance for the nurse's board and lodging in addition to her salary.

### NIGHT NURSES AT SLIGO.

There are 153 inmates at the Sligo Workhouse Hospital, and the medical officer has asked for two night nurses. His application received but little encouragement from the Guardians, who seemed to think the doctor ought to be able to persuade the day nurses to do double duty. This plan not being feasible, they directed that paupers in the house should be engaged as nurses, and rewarded by increased rations! The Sligo poor have been accustomed to pauper "nurses" at £4 per annum, and the medical officer's attempted reforms appear unlikely to find favour with the Guardians.

### FIRE AT A CHILDREN'S HOSPITAL.

Fortunately only structural damage resulted from the recent outbreak of fire at the Hospital Trousseau in Paris, all the patients being promptly removed from the scene of the catastrophe. Five hundred children are accommodated at this hospital, the doubtful and infectious cases being nursed in isolated wooden pavilions. The fire originated in the scarlet fever wards, and the patients were immediately transferred to those usually reserved for suspicious cases.

### NOTES FROM SYDNEY, N.S.W.

Sydney is supplied with good, bad, and indifferent midwifery nurses, and Dr. James Graham (at one time medical superintendent of the Prince Alfred Hospital, now member of Parliament for one of the divisions of the city of Sydney) has, during the present month, moved in Parliament, "That leave be given to bring in a Bill to promote the better training of women as midwifery nurses, and for their registration as such." The motion was agreed to, and the Bill was introduced and read the first time. A number of ladies and gentlemen met in the Town Hall, Sydney, for the purpose of discussing the best means to be adopted for establishing a public maternity hospital for Sydney. Lady Windeyer presided, and it was moved, seconded, and agreed to, "That the meeting, recognising the need for a maternity hospital in Sydney, resolves itself into a committee, with power to add to its number, for the purpose of furthering this object." At present the only important public place is in connection with the Benevolent Asylum, but the accommodation is scanty, and the building unsuitable. There are one or two other institutions that have lying-in wards in connection with them, and there are also some partly proprietary places in which maternity cases are received, but this movement is a step in the right direction.

### NURSING AT BELLEVUE, U.S.A.

It is twenty-two years since the Training School at Bellevue was organised on the lines laid down by Miss Nightingale, and 464 nurses have completed their hospital course since then. Of these some sixty are said to hold positions as heads of nursing departments in other hospitals, and the school continues to make steady upward progress.

### SHORT ITEMS.

The carnival at Aldershot has resulted in a handsome contribution to the funds of the proposed hospital which will probably be erected in the course of next year.—*Nursing Notes* for November contains interesting correspondence on various nursing matters, and amongst other articles there is a pleasant one on "The Central Home of the Queen's Jubilee Institute."—The Whitechapel Board of Guardians have adopted the recommendation of the Infirmary Visiting Committee that the period of training probationers should be increased to three years.—Miss Edith Watson, lady superintendent of the Nurses' Home, Norwich, has been presented with a charming five o'clock tea set and silver fish servers "as a token of loving esteem by the nurses of the Norfolk and Norwich staff."—A most successful concert was given last week in aid of the funds of the East Dulwich and Dulwich Nursing Society, which enjoys great popularity in the district, the services of the Queen's nurse being highly appreciated.—Miss Daniel, recently appointed head nurse at the Union Infirmary, Northampton, holds the L.O.S. diploma as well as the certificate for monthly nursing which she gained at Plaistow.

### Elementary Physiology for Nurses.

By C. F. Marshall, late Surgical Registrar Hospital for Sick Children, Great Ormond Street.

### XIV.—THE NERVOUS SYSTEM (*Concluded*).—THE SENSE ORGANS.

### Reflex Action.

The spinal cord, which we have hitherto regarded as merely a conductor of impulses to or from the brain, is really much more than this. If in a frog we destroy the brain or divide the spinal cord high up and then pinch the skin of the part (to irritate a sensory nerve) the leg is drawn up and kicked out violently. Here the will is not involved, but the spinal cord acts as a nervous centre, putting the ingoing and outgoing impulses into direct communication—in the same way that in a telephone office two people may communicate through the office without anyone in the office knowing what is going on. The purposive character of this reflex action is remarkable. If a frog under the above conditions has a drop of vinegar or other irritant placed on the thigh, it attempts to rub it off with the same leg to which the irritant is applied; and if this leg is held it will attempt to rub it off with the other leg. This is a good example of what is known as reflex action, where the essential elements are a nervous centre, a nerve conveying afferent or in-going impulses, and a nerve conveying efferent or out out-going impulses, as shown in the diagram.

FIG 9

Diagram of Reflex Action.

N.—Nerve centre. A.—Afferent nerve. E.—Efferent nerve. S.—Skin. M.—Muscle.

Reflex action may involve the brain, though not the will; for instance, winking at a flash of light, or coughing from a foreign body in the glottis. In such cases all the essentials of reflex action are present, but the will is not involved.

### The Sympathetic System.

This is an accessory nervous system in connection with the viscera and blood-vessels. It consists of branches from the spinal nerves, which form a series of nervous chains, with nerve ganglia (or aggregations of nerve cells) and large nerve plexuses in the abdomen. The vaso-motor nerves regulating the size of the blood-vessels belong to this system, and exercise a constant action over them. These, in their turn, are under the influence of the central and spinal nerves, which have the power of inhibiting them, *i.e.*, can cause the vessels to expand by withdrawing the normal restraining action of the sympathetic nerves. A good example of inhibitory action is furnished by the branch of the pneumogastric nerve which goes to the heart. This nerve when irritated causes the heart to beat more slowly, or even to stop beating altogether, a state of affairs which may occur in sudden emotion.

### The Sense Organs.

These may be divided into two sets: 1. Those concerning ourselves only, or subjective. 2. Those concerning bodies or matters outside ourselves, or objective.

*Subjective Sensations.*—These are either general, in which case they are vague, indefinable, and not easily located. For instance, the feeling of weariness, sleepiness, faintness, &c.; or special, such as headache, stomachache, toothache, &c., due to a definite disturbance of some part of the body. Subjective sensation is not easy to deal with; all we can say is that the seat of real sensation is the brain, which is proved by the effects of a blow on the head, the effects of sleep, chloroform, &c.

*Objective Sensations.*—These are more definite, and more easily and satisfactorily dealt with, because they can be referred to actual causes, which are often visible and tangible. Under this head come the impressions which we receive through the various sense organs. The purpose of sense organs is to make us acquainted with the presence and nature of objects outside ourselves, and hence are necessarily formed from the skin. It is necessary to bear this in mind, for we shall see that in all cases, however complicated they may be, they are actually found as specialised tracts of the skin, the peculiarities being due partly to modification of the skin in different places, partly to the properties of special nerves, and partly to parts of the brain to which the impulses are sent.

*The "Five Senses."*—Seeing, hearing, smelling, and tasting are tolerably definite, but it is not so with "feeling." Under this head a number of very definite and distinct sensations are usually confused, and under the sense of feeling are commonly included the senses by which we distinguish between hard and soft bodies and rough and smooth, and between hot and cold bodies. Again, the sense by which we distinguish between light and heavy weights and the sense of pain are different from each other and from the former.

*The Sense of Touch* is possessed by all parts of the body, but very unequally. It depends on the integrity of the epidermis, for if this is removed, as by a blister or a burn, contact with a foreign body causes pain and not a tactile impression. In the skin are special nerve-endings, known as tactile corpuscles, connected with the fine ramifications of the nerves. These nerve-endings are unequally distributed in different parts, and their relative amounts in different parts of the body can be tested by means of blunt compasses. At the tip of the tongue the two points of the compass can be felt as two distinct points when 1-24th of an inch apart. At the tip of the finger the distance is 1-12th of an inch: on the cheek, 1 inch; on the neck, 2 inches; while on the back the points have to be 3 inches apart before two sensations are felt (the points being felt as one sensation).

### Royal British Nurses' Association.

The Secretary of the Royal British Nurses' Association requests us to state that Miss Farquharson, of the Alfred Hospital, Melbourne, and Local Honorary Secretary of the R.B.N.A., has been appointed Matron of the Melbourne Hospital, Lonsdale Street, Melbourne. The Melbourne Hospital is a large institution, containing 282 beds and a staff of 70 nurses. Miss Farquharson was elected to the post by a large majority. Australian candidates for registration and membership of the Royal British Nurses' Association should, in future, make application for papers to this address.

### Where to Go.

Sale of Work at Trained Nurses' Club, 12, Buckingham Street, Strand, on December 5th and 6th, from half-past two to half-past nine. Entertainments on both evenings. Contributions of work and useful and ornamental articles can be sent to the Hon. Secretary. There is always a ready sale for children's clothes and for inexpensive articles suitable for Christmas presents.

### On Urine Testing.

### II.—ALBUMEN AND SPECIFIC GRAVITY.

Albumen in an otherwise normal urine is precipitated by heat. A test-tube filled about one and a-half inches deep with the sample to be examined may be heated either over a spirit lamp or by being plunged into a pan of boiling water. As it approaches boiling temperature, if albumen be present the urine will become cloudy, perhaps even very thick, and on the addition of a few drops of acetic or nitric acid this cloudiness will not disappear. There are many other ways of testing, some used for greater nicety, some for their rapidity, some because the materials are portable, but for nurses' use there is none so uniform in its effect and so reliable as that by boiling and the addition of acetic acid. Boiling by itself will not always throw down albumen, for if the urine be alkaline this alkalinity must be neutralised; on the other hand certain salts (earthy phosphates) are precipitated by boiling, but they are dissolved by the slightest acidity. Boiling, followed by the addition of acid, form therefore, when taken together, a safe test, although neither by itself is sufficient.

When the urine is thick from the deposit of urates the test will not be interfered with, as the opacity will be cleared away by the heat before the albumen is coagulated. It is well, however, to obtain as clear a specimen as possible, and if it is thick from any other cause than urates to filter it through a little white blotting paper before boiling it.

There is no accurate and easily applied test for the quantity of albumen, but a fair idea as to whether it is increasing or diminishing may be obtained by always filling the tube to the same height and letting it stand a certain number of hours (say twelve), and then noting what proportion of its height is occupied by the precipitate.

By testing both the morning and evening samples in the morning and leaving the tubes standing side by side, it is easy to determine which contains the most albumen, a point which is often somewhat important as showing the effect of exercise and food on the albuminuria.

Two other methods may be mentioned, as they are often employed by medical men. A test tube may be filled within about an inch of the top, and the flame of the spirit lamp being applied about half-way down the side of the tube, the upper portion alone may be boiled; by this means a comparison is easy between the boiled and the unboiled portion. One objection to the plan is that it is necessary to use a lamp; for if the bottom of the tube be placed in the saucepan the whole tubeful will be heated. Another is that when the acid is added it is apt to sink to the bottom of the tube and complicate the result. Testing with cold nitric acid is a very popular method, and saves a great deal of time when many specimens have to be examined. If a test tube be filled about two inches deep with the sample to be examined, and then a little strong nitric acid be allowed very gently to trickle down the side of the tube held slantingly, it will by its weight sink to the bottom, where it will lie with the urine floating upon it. Now, just at the junction where the acid and the urine come in contact a hazy line will be produced if albumen be present. This test is chiefly used where only small quantities of albumen are suspected, and is very delicate and accurate. It must be remembered, however, that while the absence of a haze denotes the absence of albumen, a haze may be also produced by other substances; so that, if it is produced, then the ordinary boiling and acid test has to be gone through. The chief use of this test is where a large number of specimens have to be examined, as it quickly tells in which cases further investigation is required. By itself it is not a complete test.

The specific gravity of urine roughly tells the amount of stuff there is dissolved in the water. Suppose one were to take a bottle of such a size that it would hold exactly 1,000 grains weight of pure water, it would clearly hold more than 1,000 grains weight of some heavier material, such as syrup or salt and water, and it would be possible to express the strength of this syrup or salt and water by the extra weight of the bottleful. This weight would be peculiar to that particular strength, and would be altered at once by the addition either of more sugar or more water to the mixture. It is therefore called its specific gravity. The ordinary specific gravity of urine is somewhere between 1,015 and 1,025, which means that it contains sufficient stuff of one sort or another, dissolved in it, to make a bulk of it equal to the bulk of 1,000 grains of pure water weigh 1,015 to 1,025 grains.

Now, the simplest way of finding out this specific gravity is by a "urinometer." We all know that if we try to swim, we sink deeper in fresh than in salt water; and so, in proportion as a fluid contains more stuff dissolved in it, or, in other words, is of higher specific gravity, a urinometer placed in it is floated up to a greater degree, and by the marks upon its stem indicates the specific gravity of this fluid. The taking of a specific gravity, then, merely involves pouring sufficient urine into the test glass, placing the urinometer in it, and noting accurately which mark on its stem remains level with the surface of the fluid in which it floats. There are one or two precautions, however, to be observed in making this observation. When warm the urine, as is the case with all fluids, is of a lower specific gravity than it is when cool. The specimen should, therefore, be allowed to cool to the temperature of the room (somewhere about 60 deg. Fahr.) before the specific gravity is taken. Then the urinometer must not be allowed to touch the side of the glass, otherwise it will not move freely. Again, the side of the stem of the urinometer may become surrounded with bubbles, which may hide the mark upon it. These may be removed with a bit of blotting paper.

If the quantity of urine available should not be enough to float the urinometer it may be diluted with exactly the same quantity of water, and the last two figures of the specific gravity then obtained be multiplied by two. Thus 1 oz. of urine at 1,030 with 1 oz. of water at 1,000 would equal 2 oz. of the mixture at 1,015, and *vice versâ*, the mixture having a specific gravity of 1,015 would show that the original fluid had one of 1,030.

The instrument, if made of glass, must not be washed in hot water.

### Appointments.

Mercers' Hospital, Dublin.—Miss Editha Manifold has been appointed Lady Superintendent of this hospital, where she has been *locum tenens* for the last two months. Miss Manifold was trained at the Meath Hospital, then did private nursing, was staff sister at the Convalescent Home, Stillargar, for six months, and took the matron's duties there in her absence. She holds good testimonials.

New Infirmary, Isleworth.—Miss Ellen E. Moriarty has obtained the appointment of Matron of the New Infirmary shortly to be opened at Isleworth. Miss Moriarty was trained in the Nightingale School, St. Thomas's Hospital, and left in 1888 to take the appointment of "home sister" at St. Marylebone Infirmary, Notting Hill. Her work there has been most valuable, and she carries with her to her new home the hearty good wishes of the whole staff.

St. George's Infirmary, Fulham Road.—Miss Adeline Griffiths has been appointed Matron at this infirmary. She was trained at Huddersfield Infirmary; was Assistant Matron at Kensington Infirmary, Matron of the Kensington Temporary Infirmary at Plaistow, and Matron since January, 1893, of the Darenth Schools. We wish Miss Griffiths success in the work, for which her previous experience is an excellent preparation. Miss Griffiths holds a variety of most satisfactory testimonials.

### Royal National Pension Fund for Nurses.

### HOW TO THANK THE MERCHANT PRINCES.

We have received a number of letters from all parts of the country with reference to our remarks under this head last week. It would be impossible to find space for all the letters, and we therefore select two which fairly represent the general opinions expressed.

Margaret Prince, District Nurse, of Burnley, writes :—

How glad I am to see that there is perhaps going to be an address of thanks given to those who have contributed so largely to the Pension Fund. It will not convey half enough gratitude to those who are so kind to us ; still, it will be a little acknowledgment of their goodness. I very often feel what a tremendous benefit the Pension Fund is to us, and wish with all my heart that I could thank somebody, but do not know where to start. It is not only the money benefit, but the kindness and thought with which everything in connection with it is done that I, and I'm sure all nurses, feel grateful for, there is such a lovely safe-and-cared-for feeling in belonging to it. An address would at least show in a small way our gratitude to those who are so good to us.

### A Generous Recognition for All.

Nurse Mary L. Downes writes from Rickmansworth as follows :—

I certainly think your letter of thanks to all the generous donors of the £23,500 is most desirable. One feels it is so difficult to make the acknowledgment such as the gift deserves, but at least it will show that the nurses are grateful for all the kindness shown and done to them. Particularly I think we ought to thank Mr. Burdett, the founder, to whose energetic interest the success of the Fund is, I fancy, mainly due. Without his kind offices these generous merchant princes would never have had their interest aroused to make such a munificent gift. His is the head and theirs the hands. In speaking first of Mr. Burdett as the founder of the Fund, I do not forget that our grateful acknowledgments are also due to the entire executive. One and all, we feel, deserve our warmest thanks, and these are not wanting, though collectively it is difficult to make them known. If I might be allowed to make a suggestion, it seems to me that we might subscribe to a present to the Princess Maud on her marriage, to show our president we appreciate her interest in us. It seems to me it would be nice to do something besides saying a few words of thanks for all the kindness shown us. It is an opportunity to make an acknowledgment to the Princess which we ought not to let slip. I heard a lady say she thought Mr. Burdett's statue should be in gold. [Has Nurse Downes ever seen the Albert Memorial ?—Ed. *T. H.*]

### Our Decision.

In compliance with the general wish we have determined to place *a book for signature at* our office, 428, *Strand*, London, W.C., and to let it lie there *for a fortnight from* Monday, *the 11th instant*, so *that nurses in London* and the immediate neighbourhood *may call and inscribe their names.* Each nurse will sign her name and policy number, and give the institution at which she is employed and the position she holds, in the column opposite her signature. Thus :—

A. B. Policy No. 40. Leeds Infirmary. Matron.
C. D. Policy No. 41. Guy's Hospital. Sister.
E. F. Policy No. 42. Nurses' Co-operation. Private nurse. And so on.

Those *nurses who reside at a distance*, and are unable to sign, *may have their names entered for them* if they will send us a *post card, not a letter*, in the above form.

The terms of the address will be published in The Hospital in due course.

### A Marriage Present for Princess Maud.

In deference to the general wish expressed by the nurses in the Pension Fund, we have consented to receive subscriptions of 1s. and upwards from every nurse who desires to participate in the gift of a wedding present to H.R.H. Princess Maud, in recognition of the nurses' gratitude to our Princess, H.R.H. the Princess of Wales. We think that it would be well if it could be arranged that every nurse should give 1s. and no more, as we feel sure this would best meet the wishes of the Royal bride and her illustrious parents. At the same time we feel that we have no right to dictate to the nurses what offering they shall individually contribute.

To save expense we must ask the nurses to be satisfied with an acknowledgment in The Hospital, all contributions received each week being published in our columns until the whole of the subscriptions are received. We must ask nurses to be kind enough to send a remittance with their promise to subscribe, and to let us have them within a month from the present date, November 6th, 1895, when the subscription list will be finally closed. An audited statement of the accounts will be published in due course, and, with the view of selecting a suitable gift, we should be glad if the contributors would indicate the form of present which commends itself to them. We propose to invite the nurse representatives on the Pension Fund Council to associate themselves with us in selecting the present in due course.

### Consideration for the Officers of the Fund.

We have been requested, in reply to numerous letters which have been sent to the office of the Pension Fund, in reference to the paragraph in the *Times* and other papers announcing the recent large addition to the Bonus Fund, to state, that there is no intention or possibility of levelling up all pensions to ten shillings a week, no matter what a nurse is entered for. The Donation Bonus Fund will be distributed at the discretion of the council, mainly, we understand, in proportion to the efforts made by each individual nurse to help herself, due regard being given to the necessities and circumstances of individual cases, when the time comes for a nurse to enter on her pension. We further desire to ask nurses to remember that the correspondence at the Pension Fund office is now so heavy—480 letters were received on a recent day—that we beg they will, as far as possible, avoid writing to the secretary, unless it is absolutely necessary for them to do so on matters of business. All the nurses can materially reduce the expenses of management and show their appreciation of the efforts that are being made for them by complying with this request.

### Notes and Queries.

The contents of the Editor's Letter-box have now reached such unwieldy proportions that it has become necessary to establish a hard and fast rule regarding Answers to Correspondents. In future, all questions requiring replies will continue to be answered in this column without any fee. If an answer is required by letter, a fee of half-a-crown must be enclosed with the note containing the enquiry. We are always pleased to help our numerous correspondents to the fullest extent, and we can trust them to sympathise in the overwhelming amount of writing which makes the new rules a necessity. Every communication must be accompanied by the writer's name and address, otherwise it will receive no attention.

### Queries.

(23) *Chiropodist*.—Could you tell me of a really good and reliable chiropodist ?—(*B*.)

(24) *Urine Testing*.—Can you tell me the best and most useful handbook on urine testing, and the price of same ?—*A Student*.

(25) *Pensions*.—Having long wished to join a pension fund I shall be particularly obliged by your giving the address of the Royal National one mentioned in last week's Hospital.—*Scotch Lassie*.

(26) *National Pension*.—Please tell me where I can get full information about nurses' pension.—*Probationer*.

(27) *Instruments*.—Is there any book which gives names of instruments needed at different operations ?—*Theatre Sister*.

(28) *Training*.—Kindly tell me whether training in a cancer and skin hospital reckons in for general training.—*H. C.*

### Answers.

(23) *Chiropodist* (*B*.).—Mrs. Staunton, 62, Mortimer Street, Cavendish Square, W., has been most highly recommended to us as quite exceptionally experienced and successful.

(24) *Urine Testing* (*A Student*).—A good book on urine testing is "A Guide to the Examination of the Urine," by J. Wickham Legg. London : H. K. Lewis. 1893. Price 3s. 6d. Nurses will probably obtain what they require from the articles now being published in the "Mirror" on "Urine Testing."

(25 & 26) *Pension Fund* (*Scotch Lassie* and *Probationer*).—Write to the Secretary, Royal National Pension Fund, 28, Finsbury-pavement, London.

(27) *Instruments* (*Theatre Sister*).—"Surgical Ward Work," by Alexander Miles, published by the Scientific Press, 428, Strand.

(28) *Training* (H. C.).—No. We thank you for your friendly letter.

### Nursing in India.

### MADRAS GENERAL HOSPITAL.

The last annual report of the nursing department at the Madras General Hospital showed that the staff last year consisted of a matron superintendent, head nurse, assistant head nurse, four first grade and twelve second grade nurses, and six pupils. These pupils would have been eligible for the diploma of general nurse at the end of the year's training but none of them remained for the complete twelve months necessary to qualify them for examination.

Systematic class instruction was given by the senior assistant surgeon and the matron superintendent, a monthly examination of pupils in the subjects thus taught being held by the resident medical officer. The course of instruction included general medical and surgical nursing, bandaging, hygiene, sick cookery, &c.

### Male Attendants.

Forty-three male ward attendants were regularly employed, the medical officer being authorised to add temporarily to their number when special cases needed extra watchers. Bi-weekly classes were held for these men by the assistant surgeon during the first year's training, one class a week being arranged for those who had from one to five years training. No examinations followed these courses in the case of the male ward attendants.

### Poor Fund.

The matron superintendent has had the administration of a Poor Fund, supplied by voluntary contributions for aiding patients with needful appliances or, when necessary, paying their railway fare.

### A New Combination.

The Madras Government has recently resolved to amalgamate the Hospital for Women and Children with the General Hospital. This arrangement will be carried out by the removal of the women and children into the quarters formerly used for the Station Hospital, which are situated conveniently near to the General Hospital. By this scheme the two establishments will be under the same management, and the nurses will benefit by a greater variety in training. Last year 37 nurse pupils were received at the Women and Children's Hospital, and it is probable that the large combined school for nurses will possess many advantages. The public nursing institution founded by Mrs. Nisbet (matron superintendent) some two years ago will also be incorporated as "The General Hospital Nursing Institute." It is proposed to keep the finances of the institute separate from those of the hospital, as the former has proved already self-supporting. A staff of six private nurses will be paid Rs. 55 and 45 per mensem, and they are to work in the hospital between their cases. The matron-superintendent is authorised to increase the nursing staff as necessity arises. Subscriptions made from time to time to the institute have resulted in nearly a thousand rupees now in hand, and Mrs. Nisbet is desirous that this should go towards a convalescent home, but whether for nurses or patients is not stated. But some suggestion on the subject will probably be made by the subscribers.

The new arrangements contemplated will so greatly increase the work of the matron-superintendent that the appointment of an assistant with full English hospital training is contemplated, and an equally competent night superintendent will doubtless be required. Four wards for female paying patients will be instituted on the same terms as those already existing for officers in the male hospital, and this is considered likely to prove a great boon to the Presidency town.

---

There are four ranks of nurses employed on the staff of the Government General Hospital, Madras, besides the probationers who enter for one year's training. They are paid at the following rates: Head nurse, Rs. 60; assistant head nurse, Rs. 50; first grade nurse, Rs. 40; second grade, Rs. 30; and probationers during their year of training receive money and clothes to the value of Rs. 15 per mensem. The nurses are provided with board, and have a home to themselves; they are expected to keep their rooms neat and clean. The probationers have apartments in the hospital itself.

All the nurses wear uniform, that of the probationers being entirely white, and our illustration shows that the costumes are very neat and English looking.

The nursing staff is under the control of the matron-superintendent, who has authority to suspend or dismiss anyone guilty of serious faults or misconduct, or who proves herself inefficient. Candidates for the posts of nurses must possess certificates of training and testimonials of good character, and they are only engaged on the distinct understanding that they will serve the hospital for two years. If they break their agreement and leave without a written permission they are fined 30 rupees, and forfeit any salary which may be due to them. The day nurses are on duty from a quarter to seven a.m. to six p.m., with an hour and a half off for meals; night nurses are on from six p.m. to a quarter to seven a.m., with one hour off.

Probationers are admitted between the ages of 18 and 30, and their training is usually considered complete at the end of one year, although it may be extended over another three months. They are afterwards expected to remain in the service of the hospital for two years, and to take such situations as hospital nurses as the matron-superintendent may offer them.

During their training a record is kept of their conduct and qualifications, and at the expiration of the time they are entered on the hospital register as certified nurses.

---

### GOVERNMENT LYING-IN HOSPITAL.

In the midwifery class last year fees were paid by eighteen European and East Indian and twenty-two native pupils, who received diplomas.

Free training was given to fifteen European and East Indian and two native women, and the Dufferin Fund and various local boards have secured training to forty more native women.

Appointment to up-country stations and dispensaries have been obtained for twelve native midwives through the Lying-in Hospital.

The nursing in the hospital is done by probationers, who only remain until they obtain their diplomas, and the annual report says: "This constant changing is not conducive to the welfare of the patients. The majority of women who join the nurse class have no natural liking for the work, and are often very ignorant, but owing to the small staff they have frequently to be placed in very responsible posts." As a result of this report the Government has agreed that a small staff of permanent nurses should be established in this hospital.

---

### A Sad Occurrence at Ormskirk.

Another proof of the perils of under-staffing workhouse infirmaries comes to us from Ormskirk, where a pauper has died from the effects of too hot a bath given by a wardsman. The nurse, to whom no blame is attached, has forty-three patients—distributed in different wards—under her care, and in the winter she has twenty more. It is impossible for any woman, however willing, to give personal supervision to every detail of the nursing of so many patients; and this unhappy accident should convince the Guardians of the dangers of employing pauper help.

### Dress and Uniforms.

By a Matron and Superintendent of Nurses.

### GOVERNMENT GENERAL HOSPITAL, MADRAS.

This charming group of nurses may well make those of us who have toiled through the burden and heat of our recent exceptional summer feel envious. Cool white Indian lawn is the regulation dress worn by all Government nurses in India, and nothing softer or brighter for the purpose could well be devised. The dress is made perfectly plain and full, buttoning in front, and clearing the ground all round. Fine white muslin is the material of which the cap is made, the shape, as shown by the illustration, being of the simplest. The straight coronet-shaped piece that surrounds the face gives a particularly becoming finish without increasing the weight. Very light and dainty also is the apron, which, however, is of the hospital shape, with a bib and straps that pass over the shoulders and fasten behind into the band at the waist. Quite in keeping with the rest of this pretty costume are the white canvas shoes, luxuries which only nurses who are on their feet all day can adequately appreciate. The coolness and brightness of shoes made in this material will go far towards alleviating the tenderness and swelling which all of us experience more or less during hot weather, raising oftentimes a formidable obstacle to the usual powers of locomotion. The stockings worn with these shoes may be either black or white, though no doubt the latter are more generally used by those who study comfort. The different grades of nurses appear to be distinguished from each other by an armlet of green satin worn round the left arm. In the case of the head nurse it bears the initials "G. H." (for Government Hospital), surmounted by the Royal crown and monogram embroidered in gold. The first grade nurse wears the armlet plain without any embroidery. The assistant nurse does not wear any armlet at all. The majority of the nurses are Eurasians, there being only two at the present time who are English. In addition to the nurses, there are male and female ward attendants who rank as assistant nurses, all of whom are natives. The men wear a picturesque costume of white drill, surmounted by a scarlet turban with a yellow border. The effect of the little bit of colour must be extremely cheerful, a fact which must never be lost sight of, even in apparently trivial matters, when attending on the sick. The lady superintendent is to be congratulated on the smart, neat, workman-like appearance of the nurses it has been our privilege to describe.

NURSES AT THE GOVERNMENT GENERAL HOSPITAL, MADRAS.

### Everybody's Opinion.

[Correspondence on all subjects is invited, but we cannot in any way be responsible for the opinions expressed by our correspondents. No communications can be entertained if the name and address of the correspondent is not given, or unless one side of the paper only be written on.]

### TRAINING FOR MALE NURSES.

"One Who Knows" writes: In answer to the query which has appeared several times in your valuable journal respecting training for male nurses, I think a young man who has nursing thoroughly at heart could not do better than join the Army Medical Staff (or, as it is now, the Army Hospital Corps). By so doing he will get training in all branches of nursing. He need only join for three years with the colours, and seven on the reserve; and if he is not afraid of work, and he gives his mind to it, he will receive a thoroughly good training instruction.

### ASYLUM WORK.

"Bonnie Dundee" writes: We have had many letters lately in The Hospital concerning the work and hours of asylum attendants. We have also read of their grievances, supposed, real, or exaggerated. Having had the happiness for many years of being an attendant on the insane both in county and private asylums, and have taken patients across the Atlantic, I cannot, after 18 years, recall much to find fault with. An attendant's is an anxious life; but it contains many bright days. For instance, when getting ready for the weekly dance, or preparing for our own theatricals, or in practising of hymns, when "Ancient and Modern," are often beautifully sung by patients. The writer of the letter signed "Lover of Justice" speaks truly, and, indeed, we have not gone back to the days described in "Hard Cash."

### MENTAL NURSING.

"A Mental Nurse" writes: As a nurse in a private asylum, I can corroborate every statement made by "An Old Nurse," and think it is quite right that the public should know what our lives really are, being placed amongst people whom the outside world are glad to get rid of. We are expected in some private asylums to exhibit all the Christian virtues, without any spiritual aid whatever, although it is acknowledged there is no work more trying to mind and body. The restlessness and irritability of those mentally afflicted tell upon the strongest constitution after a time, and when, after a long day's work, we have our night disturbed by having to wrestle with a patient for dear life, it must be owned that an asylum attendant follows a dangerous and difficult calling. The blows and bad language a nurse receives from patients are taken from whence they come; but the petty slights and insults we have to submit to from those who are supposed to know better, and figure in the world as sane beings, are not calculated to encourage us. To imagine that a woman, because she is a "mental" nurse, must be thick-skinned, ignorant, common, and without feeling is indeed a mistake. I have been nursing the insane for over eight years, and have worked in both public and private asylums, and must say it is very disheartening to feel our work is thought of so slightingly.

### FOOD IN ASYLUMS.

"One who has Charge of an Asylum Kitchen" writes: Being deeply interested in the letters appearing in The Hospital, I write from a sense of duty to correct some of the exaggerated statements made by "An Old Nurse" with regard to the quality and quantity of food supplied to the asylum nurses. I presume she has had a burnt pudding placed before her, or perhaps it was top-scorched. I will go so far as to own that occasionally a joint is a little underdone or vegetables not sufficiently well cooked. She, without mercy, publishes these details in a public paper, perhaps endangering the reputation of some most useful institution. I say shame to a nurse who would do it! She is so narrow-minded as to look grudgingly upon food supplied to deserving self-sacrificing gentlemen who hold positions as medical officers. They devote time, and even life, to a good cause. Would "An Old Nurse" expect to receive the same amount of salary as medical officers? The services of a good nurse cannot be valued too highly, but they are not equal to those of a medical man who spends years in study for his position. Can any just-minded person hold the officers responsible for occasional accidents which arise from various causes? Certainly the kitchen staff is not answerable. "An Old Nurse" fails to understand the terrible strain upon those who are responsible for the cooking for large numbers done in asylum kitchens. With what an anxious mind the cook urges on her helpers to do their best. The faces of young kitchen-maids look troubled and anxious for fear all will not be right. When dinners are passed out, then and only then, is a sigh of relief given. In large institutions there are many difficulties—steam may be troublesome, wind, rain, and frost affect fires and flues. A patient just at the most important moment of the day may become troublesome. Did "An Old Nurse" weigh all these things in her mind before giving in the public press her complaints? So many courses are open to females to earn a respectable living, that if such a state of affairs existed why did not "An Old Nurse" seek elsewhere for what she failed to obtain in her asylum? I have gained my experience in one large hospital and two rather large English asylums. I have at all times found officers—from superintendent downwards—anxious for the comforts and well-being of patients and staff. All food supplied, if plain, was good and wholesome, well cooked and neatly served, in well-appointed mess rooms. Does the "Old Nurse" fear to endanger her soul by tending with care and patience poor afflicted mortals whose parents and friends have cause to bless the hour that asylum doors have been open to receive them? I will not pretend to say that there are not many troubles to battle with. I do not for one moment doubt there being many asylum workers to bear me out in my statement.

### PRIVATE ASYLUM ATTENDANTS.

"A Private Asylum Nurse" writes: I have read with great interest the letters from asylums which one nurse had the courage to start. How true the letter of October 5th is! A lot of pity is always being given to hospital nurses, but asylum ones seem considered not worth notice. The public cannot possibly know the life of sane people in lunatic asylums. Not only are we called dreadful names, but often knocked about, and, as the nurse in her letter suggests, we are told in a polite way that we are paid for it. As regards temper we are supposed to be superhuman, and all for a pittance of £20 a year, out of which we find our own uniform. The time off duty is from 3 till 10 p.m. once a week; the rest of the week spent with patients, not only in the day, but we sleep in the same room, and attend to their wants. We have to "relieve night duty," *i.e.*, up all the day before, then night duty, and get up all our patients and stay on duty till ten the next morning; that is, twenty-seven hours straight off. Where I am now we have no nurses' sitting-room, and are not allowed to go to our bedrooms in the daytime, because they are the same as the patients'. If a nurse is off duty for her weekly eight hours, and does not feel equal to go out, she has no place where she can rest her weary mind and body. Mental work is most trying. I don't wish to say hospital nursing is not so too, but how much more interesting to be able to ease the pain of those poor suffering creatures. They are very grateful to their nurses. With mental cases we can do nothing but wait, and very few mental cases are grateful to their nurses when they get well. In fact, some of them seem only too pleased to find fault. I don't wish to be rude, but it is rather different for medical officers and matrons, who only visit the patients two or three times a day, instead of being always with insane people.

### Novelties for Nurses.

(*Continued from page* xxxvii.)

The growing inclination to have every article for hospital use made of substances which will bear the most complete disinfecting and sterilizing seems likely to end in the banishment of wood wherever possible. At the Nursing Notes Exhibition of New Appliances the newest in the way of lockers was to be seen. The accompanying drawing (No. 1) is one of these, sent by Messrs. Down Brothers. The top is a simple slab of thick glass, which can be easily removed for cleaning; the drawer is rounded. The top of the cupboard beneath is also only a sheet of glass, equally removable.

Aseptic Locker.

Every portion of this locker is therefore capable of the most complete purification. Being of galvanised iron, the drawer does not seem to open and shut quite smoothly, but where asepticism is the great point minor details must be subservient to a certain extent. Another of Messrs. Down's lockers, on the same principle, but nickel-plated and more highly finished, attracted considerable attention, though the general opinion seemed to be that metal furniture would not quickly become popular in hospital wards.

Locker from Mildmay Hospital.

We have already mentioned contributions from the Mildmay Mission Hospital. The second sketch here is of a locker sent from that institution, which is quite novel in make. It is made of hard wood, with a good-sized cupboard, acting as a seat, the back having a hinged flap, which when raised makes a convenient table. Behind this is a second little open cupboard, meant to hold such few articles as may be in constant requisition. Nurses at the Mildmay Hospital must be exceptionally ingenious, to judge by their clever adaptation of such everyday articles as Mazawattee tea tins to dainty-looking receptacles for dressings, &c., painted, or rather Aspinalled, by themselves, a distinctive colour; but the height of their achievements must be acknowledged to be the tracheotomy tent for district work, consisting of just two small clotheshorses placed round a cot or bed, making a perfect frame for the cover. These will, of course, fold up very conveniently for conveying about, and answer the purpose as well as the most elaborate frame would do.

Another simple device, the practical outcome of a nurse's experience, is the conversion of an ordinary chair into a night commode, by the introduction under the removable seat of a frame made by any carpenter and a common ware pail. Its advantages of being a cheap, hygienic, and entirely efficient substitute for a more clumsy and insanitary apparatus are apparent, and with the removal of the pail and the extra seat the chair is ready for everyday use at once.

From the London Hospital came specimens of several appliances there in use, notably an invention of "Sister Mellish," a plain long glass tube, corked at each end for keeping catheters in solution, so inexpensive a device allowing each bed to have its own tube at next to no cost. For district work this cheap and simple contrivance would be most useful. A delightful ward case, containing every requisite for a patient's toilet, consisting of a zinc or iron tray with soap, tow, ointments, back and head mixtures, glycerine, plaster, &c., is worthy of special mention, and also a first-aid case, fitted with every ordinary necessary for emergencies, for the moderate sum of 10s. Space begins to fail, but a flying mention must be made of the fascinating massage garments sent by Miss Mackenzie, and exhibited to great advantage on a miniature invalid, nestled into a tiny bed, expressly arranged for restless patients, with sheet buttoned over the quilt in a most ingenious fashion, and secured to the bed rails. A camphor jacket, too, from the Mildmay Hospital, must not be forgotten, intended for district nursing as being far more efficacious than badly-made poultices, and seldom needing changing. Nurses who have had experience of cottage poultices will appreciate its value.

One important exhibit we have left to the last, and that is peat wool, the new dressing *par excellence*. But a notice of this must be reserved for another time, and we must conclude with the mention of O'Brien, Thomas, and Co.'s baby hammock bath, consisting of a towel hammock slung by a cord across an ordinary bath with the help of hooks and rings, in which baby lies, while the mother's or nurse's hands are both left free. In cases of convulsion, or for medicated baths, this may prove very useful. We are leaving many "novelties" unnoticed for want of space, and can only conclude by warmly congratulating Miss Brierley and Mrs. Nichol, the editors of "Nursing Notes," upon the success of the exhibition, towards which the kindly assistance of many friends has contributed much.

### Death in Our Ranks.

Annie Rosser, member of the private nursing staff of the General Infirmary, Gloucester, died October 31st, 1895, of typhoid fever, beloved and regretted by all who were associated with her in her work.

"The Master calls; she tarries not,
For He has need of her above."

### Wants and Workers.

[The attention of correspondents is directed to the fact that "Helps in Sickness and to Health" (Scientific Press, 428, Strand) will enable them promptly to find the most suitable accommodation for difficult or special cases.]

Can anyone tell me where a girl of fifteen, who has four epileptic fits a week, could be taken in? Would any hospital receive her? She is fast developing into an idiot. She is one of five children, and the mother is a poor widow.

## Figures and Tables

**Fig 9 f1:**
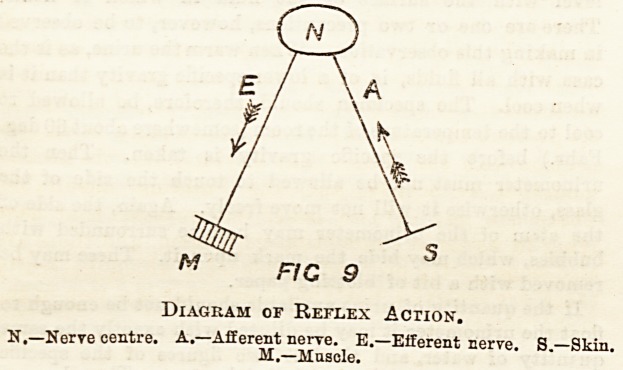


**Figure f2:**
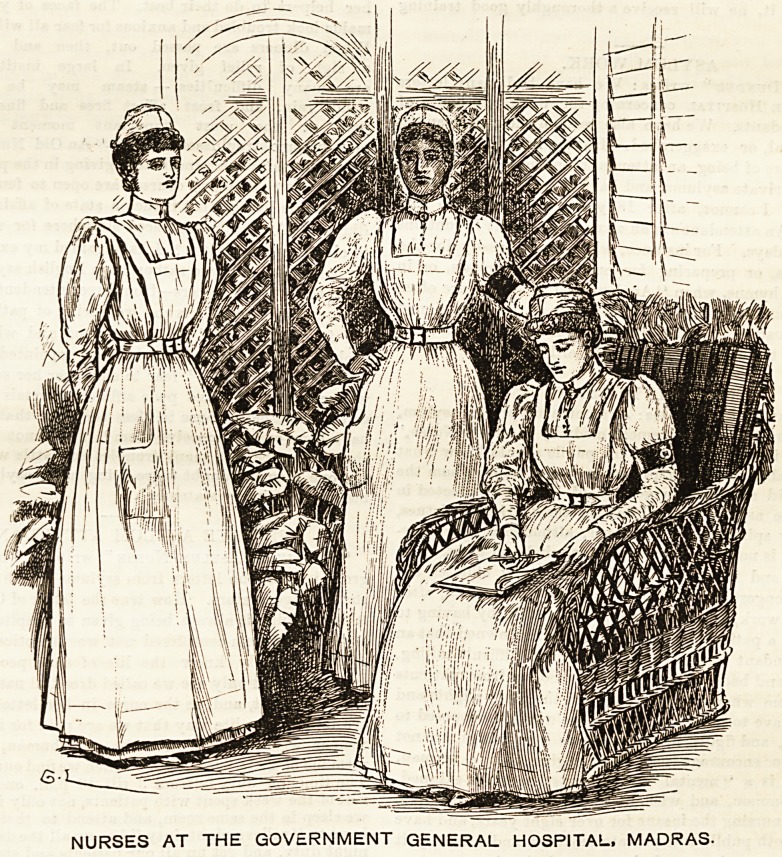


**Figure f3:**
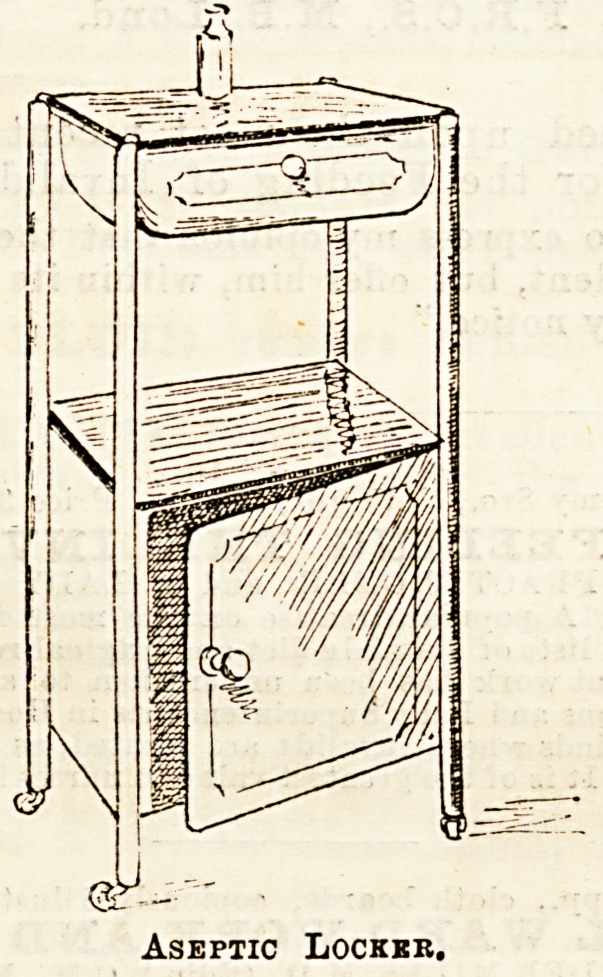


**Figure f4:**